# Mitochondrial genome-wide analysis of nuclear DNA methylation quantitative trait loci

**DOI:** 10.1093/hmg/ddab339

**Published:** 2021-11-19

**Authors:** Jaakko Laaksonen, Pashupati P Mishra, Ilkka Seppälä, Emma Raitoharju, Saara Marttila, Nina Mononen, Leo-Pekka Lyytikäinen, Marcus E Kleber, Graciela E Delgado, Maija Lepistö, Henrikki Almusa, Pekka Ellonen, Stefan Lorkowski, Winfried März, Nina Hutri-Kähönen, Olli Raitakari, Mika Kähönen, Jukka T Salonen, Terho Lehtimäki

**Affiliations:** Department of Clinical Chemistry, Fimlab Laboratories and Finnish Cardiovascular Research Center Tampere, Faculty of Medicine and Health Technology, Tampere University, Tampere 33520, Finland; Department of Clinical Chemistry, Fimlab Laboratories and Finnish Cardiovascular Research Center Tampere, Faculty of Medicine and Health Technology, Tampere University, Tampere 33520, Finland; Department of Clinical Chemistry, Fimlab Laboratories and Finnish Cardiovascular Research Center Tampere, Faculty of Medicine and Health Technology, Tampere University, Tampere 33520, Finland; Department of Clinical Chemistry, Fimlab Laboratories and Finnish Cardiovascular Research Center Tampere, Faculty of Medicine and Health Technology, Tampere University, Tampere 33520, Finland; Molecular Epidemiology, Faculty of Medicine and Health Technology, Tampere University, Tampere 33520, Finland; Molecular Epidemiology, Faculty of Medicine and Health Technology, Tampere University, Tampere 33520, Finland; Gerontology Research Center, Tampere University, Tampere 33520, Finland; Department of Clinical Chemistry, Fimlab Laboratories and Finnish Cardiovascular Research Center Tampere, Faculty of Medicine and Health Technology, Tampere University, Tampere 33520, Finland; Department of Clinical Chemistry, Fimlab Laboratories and Finnish Cardiovascular Research Center Tampere, Faculty of Medicine and Health Technology, Tampere University, Tampere 33520, Finland; Vth Department of Medicine, Medical Faculty Mannheim, Heidelberg University, Mannheim 68167, Germany; Vth Department of Medicine, Medical Faculty Mannheim, Heidelberg University, Mannheim 68167, Germany; Institute for Molecular Medicine (FIMM), University of Helsinki, Helsinki 00290, Finland; Institute for Molecular Medicine (FIMM), University of Helsinki, Helsinki 00290, Finland; Institute for Molecular Medicine (FIMM), University of Helsinki, Helsinki 00290, Finland; Institute of Nutritional Sciences, Friedrich Schiller University Jena, Jena 07743, Germany; Competence Cluster for Nutrition and Cardiovascular Health (nutriCARD) Halle-Jena-Leipzig, Jena 07743, Germany; Vth Department of Medicine, Medical Faculty Mannheim, Heidelberg University, Mannheim 68167, Germany; Competence Cluster for Nutrition and Cardiovascular Health (nutriCARD) Halle-Jena-Leipzig, Jena 07743, Germany; SYNLAB Academy, SYNLAB Holding Deutschland GmbH, Augsburg 86156, Germany; Clinical Institute of Medical and Chemical Laboratory Diagnostics, Medical University of Graz, Graz 8010, Austria; Tampere Centre for Skills Training and Simulation, Tampere University, Tampere 33520, Finland; Centre for Population Health Research, University of Turku and Turku University Hospital, Turku 20520, Finland; Research Centre for Applied and Preventive Cardiovascular Medicine, University of Turku, Turku 20520, Finland; Department of Clinical Physiology and Nuclear Medicine, Turku University Hospital, Turku 20520, Finland; Department of Clinical Physiology, Tampere University Hospital, Tampere 33520, Finland; Finnish Cardiovascular Research Center Tampere, Faculty of Medicine and Health Technology, Tampere University, Tampere 33520, Finland; Department of Public Health, Faculty of Medicine, University of Helsinki, Helsinki 00014, Finland; MAS-Metabolic Analytical Services Oy, Helsinki 00990, Finland; Department of Clinical Chemistry, Fimlab Laboratories and Finnish Cardiovascular Research Center Tampere, Faculty of Medicine and Health Technology, Tampere University, Tampere 33520, Finland

## Abstract

Mitochondria have a complex communication network with the surrounding cell and can alter nuclear DNA methylation (DNAm). Variation in the mitochondrial DNA (mtDNA) has also been linked to differential DNAm. Genome-wide association studies have identified numerous DNAm quantitative trait loci, but these studies have not examined the mitochondrial genome. Herein, we quantified nuclear DNAm from blood and conducted a mitochondrial genome-wide association study of DNAm, with an additional emphasis on sex- and prediabetes-specific heterogeneity. We used the Young Finns Study (*n* = 926) with sequenced mtDNA genotypes as a discovery sample and sought replication in the Ludwigshafen Risk and Cardiovascular Health study (*n* = 2317). We identified numerous significant associations in the discovery phase (*P* < 10^−9^), but they were not replicated when accounting for multiple testing. In total, 27 associations were nominally replicated with a *P* < 0.05. The replication analysis presented no evidence of sex- or prediabetes-specific heterogeneity. The 27 associations were included in a joint meta-analysis of the two cohorts, and 19 DNAm sites associated with mtDNA variants, while four other sites showed haplogroup associations. An expression quantitative trait methylation analysis was performed for the identified DNAm sites, pinpointing two statistically significant associations. This study provides evidence of a mitochondrial genetic control of nuclear DNAm with little evidence found for sex- and prediabetes-specific effects. The lack of a comparable mtDNA data set for replication is a limitation in our study and further studies are needed to validate our results.

## Introduction

Mitochondrial DNA (mtDNA) encodes 22 transfer RNAs, two ribosomal RNAs and 13 protein subunits of the 4 oxidative phosphorylation (OXPHOS) complexes (1). The mutation rate of mtDNA is significantly higher than that of nuclear DNA, and mitochondrial single-nucleotide polymorphisms (mtSNPs) have accumulated during evolution, dividing the human population into mitochondrial haplogroups just as populations have colonized different geographic areas of the world (2).

Most of the mitochondrial proteome is encoded by nuclear DNA, and crosstalk between mitochondria and the nucleus is essential to maintaining normal cellular function. Retrograde signals from mitochondria to the nucleus induce changes in, for example, nuclear DNA methylation (DNAm) and gene expression, which, in turn, can regulate mitochondrial functionality and metabolism (3,4). Previous cohort-level studies have shown that mtSNPs and haplogroups also associate with nuclear gene expression in peripheral blood (5,6). If the associations arose from causal relationships, they could have been mediated by epigenetic changes. This hypothesis is backed up by an *in vitro* study carried out on human retinal cell cybrids with identical nuclei but different mtDNA, which demonstrated expression differences in inflammation, angiogenesis and signaling genes between different haplogroups (7). After treatment with a methylation inhibitor, the expression levels of these genes became equivalent. Also, alterations in the global DNAm levels have been identified between haplogroups in peripheral blood (8). However, the effect of individual mtSNPs on DNAm is less known, and cohort-level association studies are lacking. Genome-wide association studies have identified numerous DNAm quantitative trait loci (9–11), but these studies have not examined the mitochondrial genome.

Epigenetics may play a role in the etiology of type 2 diabetes (T2D) mellitus (12,13), and there is evidence that epigenetic changes are likely to be an early process that may occur before the onset of T2D, i.e. during prediabetes (14). Although mtSNPs and haplogroups do not seem to be associated with prediabetes or T2D in the European population (15–17), they may have smaller consequences on a molecular level or modulate the complications of the disease (18). For example, we have demonstrated that the onset of prediabetes may lead to changes in the mitochondrial genetic control of the peripheral blood transcriptome (6). However, the crosstalk between mtDNA and the nuclear epigenome in the setting of prediabetes is not known.

In the current study, we examined the mitochondrial genetic determinants of peripheral blood DNAm obtained from 926 participants in the Young Finns Study (YFS), with an additional focus on sex- and prediabetes-specific effects. We sought replication in an independent data set consisting of 2317 individuals from the Ludwigshafen Risk and Cardiovascular Health (LURIC) study and combined the replicated results in a meta-analysis. Finally, we studied the associations of the identified CpG sites with peripheral blood gene expression to explore possible biological consequences of the differential DNAm.

## Results

### Study characteristics

Table 1 provides the basic characteristics for both cohorts. The LURIC study participants were, on average, older than the YFS participants, with a higher percentage of men and individuals with prediabetes. The proportion of current smokers was similar in both cohorts, but the percentage of never-smokers was higher in the YFS. The fraction of ex-smokers in LURIC participants was also higher in every subgroup, except among women.

**Table 1:** Basic characteristics of the YFS and LURIC cohorts; values are mean (SD) or *n* (%) for continuous and categorical variables, respectively

	All	Men	Women	Prediabetes	Controls
YFS					
No. of participants	926	401	525	263	597
Age, years	41.9 (5.1)	42.1 (5.1)	41.8 (5.1)	43.0 (5.1)	41.4 (5.0)
Women	525 (56.7)	−	−	104 (39.5)	385 (64.5)
BMI, kg/m^2^	26.6 (5.0)	27.4 (4.6)	26.1 (5.1)	28.3 (5.4)	25.5 (4.2)
Active smoker	127 (13.7)	66 (16.5)	61 (11.6)	47 (17.9)	72 (12.1)
Smokes once a week or more often but not daily	34 (3.7)	19 (4.7)	15 (2.9)	8 (3.0)	22 (3.7)
Smokes less often than once a week	36 (3.9)	16 (4.0)	20 (3.8)	10 (3.8)	21 (3.5)
Attempts to quit smoking	12 (1.3)	7 (1.7)	5 (1.0)	4 (1.5)	7 (1.2)
Has quit smoking	234 (25.3)	111 (27.7)	123 (23.4)	71 (27.0)	150 (25.1)
Has never smoked	483 (52.2)	182 (45.4)	301 (57.3)	123 (46.8)	325 (54.5)
LURIC					
No. of participants	2317	1599	718	1105	311
Age, years	62.8 (10.7)	62.0 (10.6)	64.8 (10.5)	62.0 (10.8)	57.6 (12.4)
Women	718 (31.0)	−	−	328 (29.7)	98 (31.5)
BMI, kg/m^2^	27.4 (4.1)	27.5 (3.8)	27.3 (4.7)	27.2 (3.8)	26.0 (3.8)
Heavy smokers	317 (13.7)	251 (15.7)	66 (9.2)	149 (13.5)	55 (17.7)
Light smokers	200 (8.6)	142 (8.9)	58 (8.1)	92 (8.3)	37 (11.9)
Former smokers, quit <10 years ago	319 (13.8)	254 (15.9)	65 (9.1)	154 (13.9)	43 (13.8)
Former smokers, quit ≥10 years ago	634 (27.4)	574 (35.9)	60 (8.4)	292 (26.4)	69 (22.2)
Has never smoked	847 (36.6)	378 (23.6)	469 (65.3)	418 (37.8)	107 (34.4)

### mtSNPs associated with DNAm

A total of 88 513 545 CpG–mtSNP pairs were tested in the discovery phase. The number of significant associations after accounting for multiple hypothesis testing (*P* < 7.8 × 10^−10^) was 5652, corresponding to 4618 unique CpG sites and 89 mtSNPs. The CpG sites were scattered all around the nuclear genome. A mitochondrial Manhattan plot representing the significant associations for all CpG sites is shown in Supplementary Material, Figure S1. The full list of significant associations is available as Supplementary Dataset S1. The bacon-adjusted values from the 88 513 545 CpG–mtSNP pairs yielded an estimated inflation factor (*λ*) of 1.00, which suggests minimal inflation.

**Figure 1:**
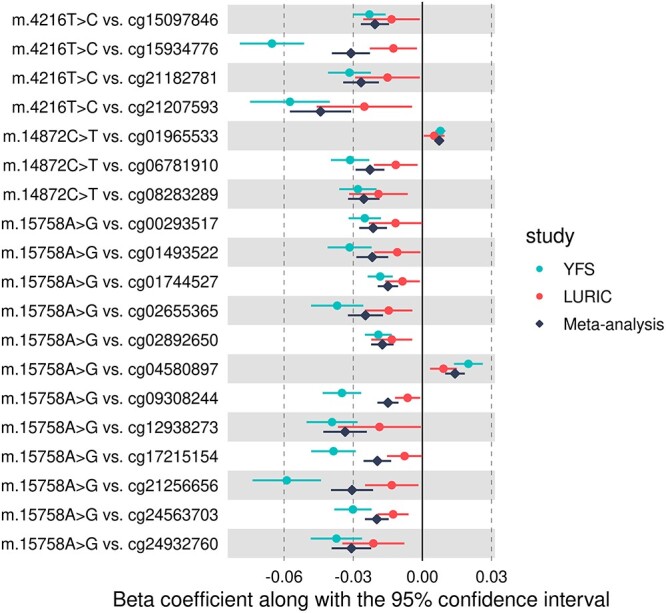
Forest plot showing the 19 nominally replicated mtSNP effects on DNAm, which also reached epigenome-wide significance in the meta-analysis.

In all, 685 CpG–mtSNP pairs that were significant in the discovery phase were available for replication, resulting in a significance level of *P* < 7.3 × 10^−5^ (0.05/685). None of the associations in the replication sample passed this threshold, even though we expected to see 228 associations to reach this *P*-value. Twenty-one associations were replicated with nominal significance (*P* < 0.05) (Table 2). At this threshold, we expected virtually all 685 associations to replicate. There was no correlation between the discovery and replication effect sizes (Pearson’s *r* = 0.06, Supplementary Material, Fig. S2), and 51% of the associations had a consistent direction of effect. The fixed-effect meta-analysis combining the nominally replicated results yielded 19 associations with epigenome-wide significance (*P* < 7.8 × 10^−10^) (Table 2 and Fig. 1).

**Table 2:** List of the 21 nominally replicated significant CpG–mtSNP associations; for all LURIC associations, the corresponding mtSNP was genotyped only with the HumanExome-12 array; 19 associations reached epigenome-wide significance in the fixed-effect meta-analysis (*P* < 7.8 × 10^−10^)

mtSNP	VAF	CpG	Chr	UCSC reference gene	YFS			LURIC			Meta-analysis		
					Effect	SE	*P*-value	Effect	SE	*P*-value	Effect	SE	*P*-value
m.499G>A	0.023	cg10790723	22	*FOXRED2*	0.030	0.005	5.1 × 10^−10^	0.002	0.001	1.7 × 10^−2^	0.002	0.001	1.1 × 10^−3^
m.4216T>C^a^	0.12	cg15934776	2	*AFF3*	−0.065	0.007	4.4 × 10^−20^	−0.013	0.005	1.6 × 10^−2^	−0.031	0.004	1.8 × 10^−13^
m.4216T>C^a^	0.12	cg21182781	1	*IARS2*	−0.032	0.005	2.6 × 10^−11^	−0.015	0.007	3.5 × 10^−2^	−0.027	0.004	1.8 × 10^−11^
m.4216T>C^a^	0.12	cg21207593	17	*LIG3*	−0.057	0.009	7.5 × 10^−11^	−0.025	0.011	1.7 × 10^−2^	−0.044	0.007	7.1 × 10^−11^
m.4216T>C^a^	0.12	cg15097846	12	*TULP3*	−0.023	0.004	1.8 × 10^−10^	−0.013	0.006	3.3 × 10^−2^	−0.021	0.003	4.4 × 10^−11^
m.14872C>T^b^	0.020	cg06781910	4	−	−0.030	0.004	1.5 × 10^−13^	−0.010	0.005	1.7 × 10^−2^	−0.023	0.003	1.1 × 10^−12^
m.14872C>T^b^	0.020	cg01965533	14	*DLST*	0.008	0.001	2.6 × 10^−12^	0.005	0.002	2.7 × 10^−2^	0.007	0.001	3.9 × 10^−13^
m.14872C>T^b^	0.020	cg08283289	13	*ZC3H13*	−0.028	0.004	1.4 × 10^−11^	−0.020	0.006	3.4 × 10^−3^	−0.025	0.003	3.5 × 10^−13^
m.15758A>G	0.014	cg09308244	2	−	−0.035	0.004	2.9 × 10^−16^	−0.006	0.003	2.0 × 10^−2^	−0.015	0.002	1.6 × 10^−10^
m.15758A>G	0.014	cg17215154	7	*IQCE*	−0.039	0.005	5.7 × 10^−15^	−0.008	0.004	4.7 × 10^−2^	−0.020	0.003	1.7 × 10^−10^
m.15758A>G	0.014	cg21256656	19	*KLK6*	−0.059	0.008	8.8 × 10^−15^	−0.013	0.006	2.5 × 10^−2^	−0.031	0.005	6.3 × 10^−11^
m.15758A>G	0.014	cg24563703	4	−	−0.030	0.004	3.0 × 10^−13^	−0.013	0.003	2.1 × 10^−4^	−0.020	0.002	6.1 × 10^−14^
m.15758A>G	0.014	cg00293517	3	−	−0.025	0.004	3.6 × 10^−12^	−0.012	0.006	4.9 × 10^−2^	−0.021	0.003	3.5 × 10^−12^
m.15758A>G	0.014	cg08251499	8	*ZFAND1*	0.032	0.005	4.0 × 10^−12^	0.002	0.001	5.8 × 10^−3^	0.003	0.011	1.0 × 10^−4^
m.15758A>G	0.014	cg12938273	22	*GNAZ; RSPH14*	−0.039	0.006	4.3 × 10^−12^	−0.019	0.009	4.3 × 10^−2^	−0.034	0.005	3.5 × 10^−12^
m.15758A>G	0.014	cg01744527	19	*SCAF1*	−0.018	0.003	4.4 × 10^−11^	−0.009	0.004	2.5 × 10^−2^	−0.015	0.002	2.9 × 10^−11^
m.15758A>G	0.014	cg24932760	17	*C17orf67*	−0.037	0.006	5.6 × 10^−11^	−0.021	0.007	2.0 × 10^−3^	−0.031	0.004	2.3 × 10^−12^
m.15758A>G	0.014	cg01493522	13	−	−0.032	0.005	9.5 × 10^−11^	−0.011	0.005	3.4 × 10^−2^	−0.022	0.004	7.7 × 10^−10^
m.15758A>G	0.014	cg02892650	16	*LRRC36*	−0.019	0.003	1.5 × 10^−10^	−0.013	0.005	3.5 × 10^−3^	−0.017	0.003	3.5 × 10^−12^
m.15758A>G	0.014	cg02655365	6	*STK38*	−0.037	0.006	1.7 × 10^−10^	−0.015	0.005	5.5 × 10^−3^	−0.025	0.004	2.2 × 10^−10^
m.15758A>G	0.014	cg04580897	3	*SNORA6; RPSA*	0.020	0.003	4.1 × 10^−10^	0.009	0.003	2.1 × 10^−3^	0.014	0.001	7.7 × 10^−11^

Abbreviations and definitions: VAF, variant allele frequency in the discovery sample; Chr, chromosome; SE, standard error.

^a^Tagged mtSNP m.15452C>A used in LURIC.

^b^Tagged mtSNP m.2259C>T used in LURIC.

#### Sexual dimorphism

In the YFS, fixed-effect meta-analysis revealed significant differences in the effect sizes between the sexes for 664 CpG–mtSNP pairs, corresponding to 35 unique mtSNPs and 621 CpG sites, nine of which were located on the X chromosome (Supplementary Material, Dataset S2). Inflation of the results was minimal in both the male- and female-specific analyses (*λ* = 1.00 for both sexes). In the LURIC study, 135 of the 664 associations were available for replication. In all, 46% of the associations had a consistent direction of effect and there was no correlation of effect sizes between the discovery and replication cohorts (Pearson’s *r* = −0.08, Supplementary Material, Fig. S3). None of the 135 associations exhibited sex-specific heterogeneity with *P* < 3.7 × 10^−4^ (0.05/135) or with *P* < 0.05.

#### Prediabetes-specific effects

In the discovery phase, 483 CpG–mtSNP pairs demonstrated a significant difference in the effect sizes between individuals with prediabetes and controls, corresponding to 470 unique CpGs and 26 mtSNPs (Supplementary Material, Dataset S3). No inflation was observed (*λ* = 1.00 for both groups). For replication, 113 CpG–mtSNP pairs were available, none of which were replicated with heterogeneity *P* < 4.4 × 10^−4^ (0.05/113) or with *P* < 0.05. No correlation of effect sizes between the two cohorts was observed (Pearson’s *r* = −0.04, Supplementary Material, Fig. S4), and 50% of the associations had a consistent direction of effect.

### Haplogroups associated with DNAm

The haplogroup frequencies and the corresponding phenotype characteristics of both cohorts are shown in Supplementary Material, Table S1. In both cohorts, the most common major haplogroup was H. In the discovery phase, a haplogroup-based analysis identified 142 significant associations (Supplementary Material, Dataset S4) with minimal inflation (*λ* = 0.99). The differentially methylated CpG sites were associated with six haplogroups: I (58.5% of the associations), X (22.5%), W (9.2%), K (4.9%), T (4.2%) and J (0.7%).

**Figure 2:**
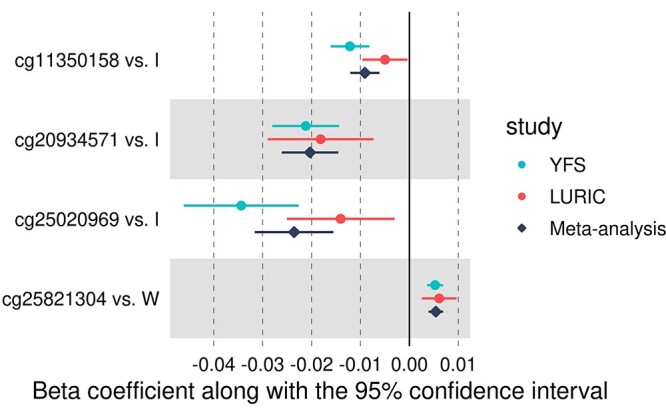
Forest plot showing the four nominally replicated haplogroup effects on DNAm, which also reached epigenome-wide significance in the meta-analysis. In all associations, haplogroup H was used as the reference haplogroup.

Twenty-two of the CpG sites that showed differential methylation in the YFS were not available in LURIC, leaving 120 CpG–haplogroup pairs for replication and setting the significance threshold at *P* < 4.2 × 10^−4^ (0.05/120). None of the associations in the LURIC survived this threshold; 15 associations were expected to reach this level. Six associations were nominally replicated, with the strongest association corresponding to *P* = 8.2 × 10^−4^. At a nominal threshold of *P* < 0.05, we expected all associations to replicate. There was a weak correlation between the discovery and replication effect sizes (Pearson’s *r* = 0.23, Supplementary Material, Fig. S5), with 62% of the associations showing a consistent direction of effect. Four associations were significant in the meta-analysis (*P* < 1.0 × 10^−8^) (Table 3 and Fig. 2).

**Table 3:** List of nominally replicated CpG-haplogroup associations; four associations were significant in the meta-analysis (*P* < 1.0 × 10^−8^)

Haplogroup	CpG	Chr	UCSC reference gene	YFS			LURIC			Meta-analysis		
				Effect	SE	*P*-value	Effect	SE	*P*-value	Effect	SE	*P*-value
W	cg25821304	17	*RNF135*	0.005	0.001	6.1 × 10^−10^	0.006	0.001	8.2 × 10^−4^	0.005	0.001	2.2 × 10^−12^
I	cg20934571	13	*CARKD*	−0.021	0.003	1.3 × 10^−9^	−0.018	0.006	1.0 × 10^−3^	−0.020	0.003	5.9 × 10^−12^
I	cg11350158	2	*LRP1B*	−0.012	0.002	1.7 × 10^−9^	−0.005	0.002	3.3 × 10^−2^	−0.009	0.001	2.6 × 10^−9^
I	cg24280540	8	−	0.030	0.005	4.6 × 10^−9^	0.009	0.004	1.8 × 10^−2^	0.017	0.003	5.7 × 10^−8^
I	cg04933492	16	−	−0.028	0.005	5.8 × 10^−9^	−0.008	0.004	4.4 × 10^−2^	−0.015	0.002	2.7 × 10^−7^
I	cg25020969	7	*MAD1L1*	−0.034	0.006	9.8 × 10^−9^	−0.014	0.006	1.3 × 10^−2^	−0.024	0.004	9.3 × 10^−9^

### Expression quantitative trait methylation analysis

Overall, the replication phase identified 27 CpGs that showed differential methylation between mtSNPs or haplogroups. We considered genes ±1 Mb from each CpG site and tested 890 gene–CpG combinations for differential expression. Two associations were significant after correction for multiple testing: inverse associations were observed for cg25020969 (which showed lower methylation levels in haplogroup I) and probes ILMN_1681674 and ILMN_2358069, both at the *MAD1L1* gene (effect estimate: −4.63 and − 3.68, standard error: 1.05 and 0.91, *P*-value: 1.2 × 10^−5^ and 5.9 × 10^−5^, respectively).

## Discussion

The aim of the current study was to examine whether mtDNA variants and haplogroups associate with peripheral blood DNAm. Although previous studies have investigated the effect of haplogroups in cybrid cell lines or by using smaller sample sizes, the present study is, to the best of our knowledge, the first to examine the associations of mtSNPs on a cohort level and the largest to investigate the haplogroups’ effects. In the discovery analysis of a Finnish population-based cohort, we identified numerous significant associations suggesting mitochondrial genetic control of DNAm and even pinpointed associations showing sex- and prediabetes-specific heterogeneity. Twenty-seven associations were nominally replicated in a German hospital-based cohort. The nominally replicated results were included in a joint meta-analysis of the two cohorts after which 23 associations remained significant. We were able to attempt replication only for approximately 15 – 30% of the mtSNP-based associations from the discovery phase, mainly owing to different mtDNA genotyping methods. We observed significantly lower replication rates than predicted even when we used a relatively liberal replication threshold (*P* < 0.05). The lack of replication was not explained by the winner’s curse. Study-specific heterogeneity owing to different cohort characteristics may have had a major impact on replication. In addition, most of the effects identified in the YFS population may simply not be present among the LURIC study participants.

The most promising association was the one between haplogroup W and CpG site cg25821304, mapping to the gene *RNF135* (Table 3)*.* It was significant in the discovery phase, reached borderline significance in replication and was significant in the meta-analysis. This CpG site did not show significant mRNA transcript associations, complicating the interpretation of the functional relevance. It has been documented that mitochondria are important participants in innate immune responses to pathogens and cellular damage and that also mtDNA variation could influence those immune response pathways (19). Our finding provides suggestive evidence for this since the protein encoded by the *RNF135* gene is involved in the evoking of innate immunity against RNA virus infections (20).

Of all the 27 identified CpG sites, 2 were significant in the expression quantitative trait methylation analysis. These associations, both corresponding to cg25020969, were not surprising, as the target gene of this CpG site and the two associated transcripts were the same, *MAD1L1.* It should be noted, however, that rather than being a strict dynamic mechanism for regulating gene expression, DNAm changes can also serve as a long-term memory of previous gene expression decisions that were mediated by transcriptional factors that might no longer be present in the cell (21).

The retrograde signals through which mitochondria affect nuclear DNAm appear to be mediated by tricarboxylic acid (TCA) cycle metabolites (22,23). *α*-Ketoglutarate (*α*-KG) serves as a cofactor for ten-eleven translocation hydroxylases (TET1-3) involved in DNA demethylation, whereas fumarate and succinate inhibit the TET enzymes. Even though the enzymes involved in the TCA cycle are not encoded by mtDNA, the TCA cycle is in constant feedback with the OXPHOS complexes, providing a plausible link between mtDNA variation, TCA metabolites and DNAm (22). In addition, experimental findings have directly coupled mtDNA variation with TCA metabolites and histone methylation (24). The three DNA methyltransferases (DNMT1, DNMT3A and DNMT3B) use S-adenosyl methionine (SAM) as a methyl donor. Although SAM is generated by coupling the methionine and folate cycles in the cytosol, these cycles are dependent on intermediate mitochondrial metabolism and ATP, and therefore, mtDNA variation may affect the function of DNMTs (3). To support these hypotheses, experimental findings on mouse embryonic stem cells have shown that mitochondrial haplogroups modulate the key regulators of both DNAm and demethylation, DNMT1 and TET1, leading to haplogroup-specific DNAm and gene expression patterns (25).

The association between haplogroup I and cg20934571 (Table 3) may represent the retrograde response aiming to regulate mitochondrial function. The CpG annotates to NADPHX dehydratase (*NAXD*, also known as *CARKD*), and the protein product may be targeted to the mitochondria. The protein catalyzes the repair of NADPHX, a damaged form of reduced nicotinamide adenine dinucleotide phosphate (NADPH) (26), and mitochondrial NADPH plays a critical role in protecting the cells against mitochondrial oxidative stress (27). Based on this, it could be suggested that mutations defining haplogroup I result in disturbances in NADPH homeostasis, which leads to compensatory epigenetic changes. Another interesting association is between variant m.14872C>T (in the *MT-CYB* gene, a subunit of OXPHOS complex III) and cg01965533 (Table 2), which annotates to dihydrolipoamide succinyltransferase (*DLST*). The protein product of *DLST* is a subunit of the *α*-KG dehydrogenase complex, which is a key control point in the TCA cycle (28). Even though complex III is not directly coupled to the TCA cycle, the identified association could result from mitochondrial–nuclear communication, such as alterations in the electron transport chain that are compensated by epigenetic changes. However, these speculations are purely hypothetical and these two CpGs sites did not associate with mRNA transcripts.

We did not observe differential methylation at CpG sites mapping to the genes that showed mtSNP- or haplogroup-specific transcriptome profiles in Kassam *et al*. (5) or in our previous study (6). In addition, variant m.3480A>G was strongly associated with nuclear DNA transcripts in both of these previous studies but was not significantly associated with DNAm at any stage in the present study. This suggests that, if there is a causal relationship between mtSNP m.3480A>G (and the mtSNPs tagged by it) and peripheral blood transcriptomics, the expression regulatory mechanisms are not mediated by changes in DNAm.

In a study using articular cartilage cells (29), haplogroup J was associated with differentially methylated CpG sites when compared with haplogroup H. We could not validate these results, as only one CpG site was differentially associated with haplogroup J in the discovery phase and none during replication. The present and the aforementioned study had different sample sizes and utilized different DNAm arrays; we examined approximately 30 times more CpG sites and haplogroup H or J carriers. However, the DNAm profiles in peripheral blood do not necessarily reflect similar methylation changes in other tissues (30).

The meta-analysis showed no evidence of prediabetes-specific heterogeneity. Further studies using more homogeneous cohorts or larger sample sizes should be conducted to gain more insight into the interplay between mtDNA variation and DNAm in the setting of prediabetes.

The maternal inheritance of mtDNA could create male–female asymmetry in the consequences of mtDNA mutations since mtSNPs that only affect males will not be subject to natural selection (31). This hypothesis has been tested in *Drosophila melanogaster* in which a strong effect of mtSNPs on gene expression was observed only in males, while the mitochondrial effect in females was negligible (32). In humans, there is no evidence of sex-specific mitochondrial genetic control of peripheral blood gene expression (5,6). Even though sex-specific DNAm patterns have been demonstrated in peripheral blood (33,34), our results imply that, similarly to gene expression, mtDNA variation has the same genetic effect on peripheral blood DNAm in both sexes.

### Strengths and limitations

The present study has strengths and limitations that warrant consideration. The variants in the YFS were obtained through next-generation sequencing, which allowed us to study a broad range of mtDNA variants. Genotyping a part of the LURIC participants with two different microarrays increased the quality of haplogroup assignment. We were also able to verify the self-reported smoking status with the cotinine measurements in the LURIC study. The discovery analyses were adjusted for bias and inflation using a state-of-the-art method that was specifically developed for epigenome-wide association studies, which maximizes power while properly controlling the false-positive rate (35). Still, it is important to note that, as with any (epi)genome-wide association study, it is possible that some of the identified associations represent false positives.

The main weakness was the lack of a comparable mtDNA data set for replication, as many of the sequenced mtSNPs in the YFS were not genotyped in the LURIC. For some mtSNPs, replication was sought by using a tagged mtSNP, which could have resulted in false-positive or false-negative replications. The smoking status in the YFS was only self-reported and was not verified by cotinine measurements. Finally, it should be highlighted that the YFS is a population-based study, whereas the LURIC participant pool mainly consists of older patients referred to coronary angiography. As DNAm variation has been associated with age numerous times, the difference in the ages between the participants of the two cohorts may have affected the results. Also, other confounding factors owing to contrasting participant characteristics may have yielded an effect on the results since socioeconomic status (36) and lipid composition (37), for instance, have independent effects on DNAm.

## Conclusion

This study provides evidence of a mitochondrial genetic control of autosomal DNAm, with little evidence found for sex- and prediabetes-specific effects. The functional relevance of the identified associations remains unclear. Further replication studies, preferably using sequencing data and more homogeneous study groups, should be conducted to thoroughly establish the mitochondrial genetic determinants of DNAm.

## Materials and Methods

### Study populations

The YFS (http://youngfinnsstudy.utu.fi) is a Finnish longitudinal population study on the evolution of cardiovascular risk factors from childhood to adulthood (38). We utilized data from two follow-ups conducted in 2007 and 2011, including 2204 and 2060 participants, respectively. Phenotypic information and DNAm data were collected in 2011, and mtDNA data were obtained from the 2007 follow-up samples. The study was approved by the ethics committee of the Hospital District of Southwest Finland, and the study protocol of each study phase corresponded with the proposal of the World Health Organization.

The LURIC study consists of 3316 patients of German ancestry who underwent coronary angiography between 1997 and 2000 at a tertiary care center in Southwestern Germany (39). The clinical indications for angiography were chest pain or non-invasive tests that were consistent with myocardial ischemia. Patients with any acute illness other than acute coronary syndrome, any predominant non-cardiac disease and/or a history of malignancy within the past 5 years were excluded from the study. The study plan was approved by the ethics committee of the State Chamber of Physicians of Rhineland-Palatinate.

All participants in both cohorts gave their written informed consent, and the studies were conducted in accordance with the Declaration of Helsinki.

### DNAm assessment and quality control

In both cohorts, genomic DNA was extracted from peripheral whole blood samples by standardized methods. DNAm levels were quantified using the Illumina Infinium MethylationEPIC BeadChip according to the manufacturer’s protocols. The array covers over 850 000 methylation sites across the nuclear DNA.

In the YFS, DNAm data were processed using the minfi Bioconductor package in R (40). All analyzed samples had a sum of detection *P*-values across all probes of <0.05. The log2 -median of methylated and unmethylated intensities among the analyzed samples clustered within the default threshold (10.5) of the getQC function in minfi. Samples for which the self-reported sex did not match with the predicted sex obtained with the getSex function in minfi were excluded. Background subtraction and dye-bias normalization were performed via the noob method (41), and stratified quantile normalization was performed using the preprocessQuantile function, both implemented in minfi. Probes with a detection *P*-value of >0.01 in 99% of the samples and cross-reactive probes (42,43) were excluded from the analysis. Probes with SNPs were removed using the dropLociwithSnps function in minfi. After quality control, the total number of autosomal CpGs was 769 683 in 1529 samples. In addition, the sex-specific analyses included 17 334 X-chromosomal CpGs.

In the LURIC study, quality control was implemented using the CPACOR pipeline (44), excluding samples with a call rate of ≤ 0.95 and those that showed sex discordance. CpGs located in close proximity (1–2 bp) to a genetic polymorphism in the European population with a frequency of >0.01% as well as cross-reactive probes and probes with a detection *P*-value of >0.05 in at least 1% of the samples were removed using the rmSNPandCH function in the DMRcate package (45), followed by quantile normalization. A total of 795 619 autosomal and 18 138 X-chromosomal CpGs from 2423 samples were included in further analyses.

Beta values [ranging between 0 (no methylation) and 1 (full methylation)] were calculated according to the equation *b* = *M*/(*M* + *U* + 100), where *M* and *U* denote the methylated and the unmethylated signals, respectively.

### mtDNA sequencing and data processing in the YFS

In the YFS, mtSNPs were determined by next-generation sequencing. The pipeline has been described in detail earlier (46). In brief, mtDNA was amplified from genomic DNA samples (*n* = 1807) and was sequenced with the Illumina HiSeq system. Reads from all samples that achieved any mean bait coverage (*n* = 1658) were aligned with the revised Cambridge Reference Sequence (1) and were analyzed using Mutserve version 1.2.1, a stand-alone version of the web tool mtDNA-Server (47), with the default parameters. Variants overlapping with any mtDNA-like sequence in the nucleus (NUMTs) were excluded. The list of NUMTs insertions was based on the work by Dayama *et al*. (48). The minimum heteroplasmy level was set at 0.05—we defined sites with a heteroplasmy level below this threshold as homoplasmic wild-type alleles and those with a heteroplasmy level >0.95 as homoplasmic variants. Mutserve identified variants in 1365 different nucleotide positions from 1657 samples. We required each sequenced sample to have an overall mean coverage of ≥30 and 1531 samples survived this threshold. The average coverage across all samples was 536.

Samples without complete phenotype, DNAm and mtDNA data were excluded after which 926 samples (525 women and 401 men) remained for further analysis, with 241 mtSNPs having an allele frequency of ≥0.01. Heteroplasmic variants were excluded owing to their low number. To reduce the computational effort, we selected a set of 37 tagging mtSNPs (Supplementary Material, Table S2) that captured 126 other mtSNPs with a linkage disequilibrium of *r*^2^ ≥ 0.8 by using Tagger (49) and HaploView (50). Seventy-eight mtSNPs were not tagged by any another variant, which resulted in 115 mtSNPs to be included in the association analyses.

Haplogroups were determined by using HaploGrep version 2.2.0 (51) (Phylotree build 17) (52). For association testing, the haplogroups were assigned to major haplogroups. Haplogroups with a frequency of <0.01 and samples whose haplogroup quality score was <0.90 were excluded, leaving 863 samples with nine haplogroups for the haplogroup–CpG analysis.

### mtDNA genotyping and data processing in the LURIC study

Genomic DNA was extracted from peripheral blood, and the mtSNPs were genotyped using the Illumina HumanExome-12 version 1.2 BeadChip (*n* = 1981) and the Illumina 200 k MetaboChip (*n* = 3150) microarrays. Samples with a call rate of <0.95, sex mismatch and cryptic relatedness (pi-hat > 0.2) were removed using PLINK version 1.90b6.21. Variants with an allele frequency of <0.005 and a call rate of <0.95 were also excluded. Heterozygous genotypes possibly owing to mitochondrial heteroplasmy were coded as missing.

After quality control and the exclusion of samples with missing phenotype, DNAm or mtDNA data, 1456 and 2290 samples from the HumanExome-12 and MetaboChip arrays, respectively, were available for further analyses. Of the variants genotyped with these arrays, 53 HumanExome-12 mtSNPs and 42 MetaboChip mtSNPs had an allele frequency of ≥0.005. Most of the genotyped individuals (*n* = 1429) were present in both arrays, and the total number of individuals was 2317 (718 women and 1599 men).

Haplogroups were assigned by applying HaploGrep separately to the two genotyping batches by applying the ‘–chip’ parameter. We included haplogroups based on two criteria: (1) a quality score ≥0.90 in at least one genotyping batch, or (2) a quality score of ≥0.80 and the same major haplogroup assigned in both arrays. This resulted in 998 samples to be included in the haplogroup–CpG analysis.

### Definition of clinical variables

Height and weight were measured, and body mass index (BMI) was calculated as weight in kilograms divided by height in meters squared. Sex was self-reported. In the YFS, the smoking history of the participants was self-reported and was classified into six categories based on smoking frequency (active smoker or at least once a day, once a week or more often but not daily, less often than once a week, attempts to quit, has quit and has never smoked). In the LURIC study, smoking status was also self-reported but was additionally verified by the measurement of serum cotinine concentration. A commonly used cut-off to define active smoking is 15 μg/l (53), and we used this value to reclassify self-reported non- or ex-smokers as active smokers. Participants were categorized into five groups: heavy smokers (defined as smoking ≥20 cigarettes per day), light smokers, former smokers who quit smoking <10 years ago, former smokers who quit smoking >10 years ago and never-smokers.

The classification of prediabetes was based on the criteria of the American Diabetes Association (54). Venous blood samples were drawn after an overnight fast for the determination of serum glucose and glycated hemoglobin A_1c_ (HbA1c). Individuals with prediabetes were defined as having a fasting plasma glucose (FPG) level of 5.6–6.9 mmol/l, a 2-h plasma glucose level of 7.8–11.0 mmol/l during a 75-g oral glucose tolerance test (OGTT), or an HbA1c level of 39–47 mmol/mol without a diagnosis of T2D. The diagnosis of T2D included individuals with an FPG level of ≥7.0 mmol/l, a 2-h glucose level of ≥11.1 mmol/l during an OGTT or an HbA1c level of ≥48 mmol/mol, or those who reported using oral glucose-lowering medication or insulin (but had not reported having type 1 diabetes) or who reported having been diagnosed with T2D by a physician. Those diagnosed with type 1 diabetes were also ruled out. OGTTs were performed only for the LURIC participants.

### Discovery analysis in the YFS

Differentially methylated CpG loci for mtSNPs were identified using a linear regression model where the methylation beta values were modeled as a linear function of the presence (coded as 1) or absence (coded as 0) of the variant allele using the lm function in R. The model involved adjustment for age, sex, BMI, smoking status, white blood cell type proportions, methylation batch effects and principal components (PCs) derived from the mtDNA data. The fraction of white blood cells (CD8T, CD4T, NK cells, B cells, monocytes and granulocytes) was estimated through the reference-based Houseman method (55) using the estimateCellCounts function in the minfi package (40). Methylation batch affects were addressed by including the first five PCs of array control probes in the regression models. PC analysis was performed on all mtDNA genotypes that passed quality control using the logisticPCA package (56). The use of mitochondrial PCs as covariates has been demonstrated to be a robust method to adjust for population stratification in genetic association studies. In addition, the use of mitochondrial PCs effectively removes false-positive associations but does not cause a loss of power in detecting true associations (57,58). All CpG–mtSNP analyses were adjusted for the first six mitochondrial PCs. CpG loci were considered differentially methylated if they reached a Bonferroni-corrected *P*-value of 7.8 × 10^−10^ (9 × 10^−8^/115) based on the number of independent tests in a whole blood EPIC array (59) and the number of mtSNPs.

#### Differential methylation between haplogroups

We applied a similar linear model to flag CpG sites for those showing differential methylation between the nine haplogroups. We selected the most common haplogroup H to be the reference to which other haplogroups were compared. Mitochondrial PCs were excluded from the covariates since the haplogroups are strongly correlated with the mitochondrial PCs. Significance was defined as *P* < 1.0 × 10^−8^ (9 × 10^−8^/9).

#### Sex- and prediabetes-specific analyses

The sex-specific effects of mtSNPs on methylation beta values were tested by applying the same linear model as described above to males and females separately. Differences in effect sizes were compared by applying a fixed-effect inverse variance-weighted meta-analysis model to each CpG–mtSNP pair by pooling the effect estimates and standard errors from males and females in Genome-Wide Association Meta-Analysis (GWAMA) software version 2.1 (60). Heterogeneity was examined by calculating the sex-heterogeneity *P*-value (61). A significant *P*-value suggests that there is a significant difference in effect sizes between the sexes. A minimum variant allele count of 10 in both sexes was required, which resulted in 63 mtSNPs to be included and a significance threshold of 1.4 × 10^−9^ (9 × 10^−8^/63).

The effect of prediabetes on the association between mtSNPs and DNAm was studied similarly by applying the linear model separately to individuals with prediabetes and normoglycemic controls and by pooling the results in GWAMA. The number of mtSNPs was 47, and significance was defined as *P* < 1.9 × 10^−9^ (9 × 10^−8^/47).

#### Control for bias and inflation

We corrected the effect estimates, their standard errors and the corresponding *P*-values for bias and inflation using the R package bacon (35), and all reported results are bacon-corrected. We used the inflation function in the same package to compute the inflation factor *λ* for each association analysis from all CpG–mtSNP/haplogroup pairs that were analyzed. The regime of minimal inflation is *λ* < 1.14 (35).

### Replication in the LURIC study

We sought replication in the LURIC study by applying the same linear regression models as in the discovery phase. We included variants with an allele frequency of >0.005, or a minimum variant allele count of five in the sex- and prediabetes-specific analyses. If a tagging mtSNP from the discovery sample was not genotyped in the replication sample, an mtSNP for replication was searched from the tagged mtSNPs. If several tagged mtSNPs were genotyped, linear regression was performed on all tagged variants and the sentinel mtSNP with the smallest association *P*-value was used.

Associations were considered fully replicated if the replication *P*-value from the linear regression model fell below a Bonferroni-corrected *P*-value of 0.05/*n*, with *n* being the number of significant associations in the discovery study covered in the replication sample. For nominal replication, the *P*-value threshold was set at 0.05. We also required consistent effect directions across both cohorts and in males/females and individuals with/without prediabetes. The two Illumina microarrays were analyzed separately, including the 12 overlapping mtSNPs present in both arrays, thus providing the opportunity of validation in the case of significant results. Associations with *P* < 0.05 in one genotyping batch and with *P* > 0.05 in another batch were not regarded as replications.

We benchmarked the observed replication rates for general mtSNP and haplogroup analyses by calculating the expected degree of replication. First, we used a false-discovery rate inverse quantile transformation to correct the effect sizes for the winner’s curse (62) and also took into account the lower number of mtSNPs available in the replication cohort. Second, we calculated the expected number of associations meeting the Bonferroni-corrected replication threshold by using the method described in Okbay *et al*. (63)

Finally, we performed a fixed-effect inverse variance-weighted meta-analysis of the replicated associations by combining the effect estimates and standard errors from the discovery and replication cohorts with the GWAMA software. An association was considered to be significant if the meta-analysis *P*-value fell below the significance threshold used in the corresponding discovery analysis. The inverse variance-based method compensates for the varying number of samples in the cohorts by allowing larger studies to have more weight in the analysis (64).

### Expression quantitative trait methylation analysis

To gain insight into whether our association data were connected to biological processes, we examined the associations between peripheral blood genome-wide transcriptomics and the differentially methylated CpG sites identified in the replication phase. Gene expression and DNAm data were available for 1364 YFS participants. The expression data were analyzed using the Illumina HumanHT-12 v4 Expression BeadChip. The procedures have been described previously (6).

CpGs were regressed against cell count proportions and the first 30 PCs of the array control probes. Similarly, the 19 637 transcription probes were regressed against the first 20 PCs derived from the expression data. For each CpG site, expression probes within a 2 Mb window (± 1 Mb) were included. Linear regression was applied between the residuals from the CpG regression (explanatory variable) and the expression probe residuals (dependent variable). The model was additionally adjusted for age, sex and BMI. The *P*-value for statistical significance was defined as 0.05 divided by the number of combinations between CpGs and genes.

*Conflict of Interest statement.* None declared.

## Supplementary Material

Supplementary_figures_ddab339

Supplementary_tables_ddab339

Supplementary_Data_Set_1_Variants_ddab339

Supplementary_Data_Set_2_Sex_Heterogeneity_ddab339

Supplementary_Data_Set_3_Prediabetes_Heterogeneity_ddab339

Supplementary_Data_Set_4_Haplogroups_ddab339

## Data Availability

The data sets generated and analyzed during the current study comprise health-related participant data, and their use is therefore restricted under the regulations concerning professional secrecy (Act on the Openness of Government Activities, 612/1999) and sensitive personal data (Personal Data Act, 523/1999, implementing the EU data protection directive 95/46/EC). Owing to these legal constraints, the individual-level data cannot be stored in public repositories or otherwise made publicly available but are, however, available from the authors upon a reasonable request.
