# Supplementary material for: Mitochondrial genome-wide analysis of nuclear DNA methylation quantitative trait loci
Source: Hum Mol Genet. 2021 Nov 19;31(10):1720–32. doi: 10.1093/hmg/ddab339 (PMC9122653; doi:10.1093/hmg/ddab339)
Supplement: Supplementary_figures_ddab339 [file supplementary_figures_ddab339.pdf]

Mitochondrial genome-wide analysis of nuclear DNA methylation quantitative trait loci

Supplementary figures

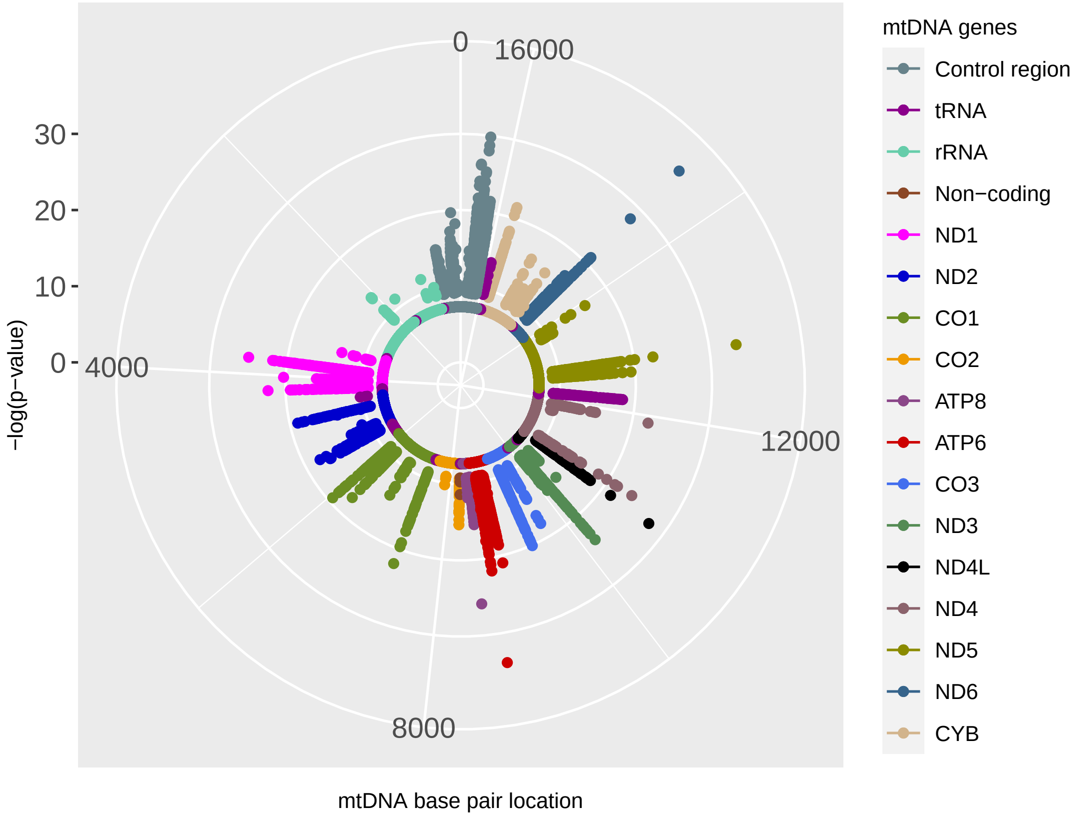

Figure S1: Mitochondrial Manhattan plot representing the significant hits ($p < 7.8 \cdot 10^{-10}$) for all CpG sites from the discovery phase. Each dot represents an mtSNP association with a CpG site, and the coloured circle represents the mitochondrial genome, both colour-coded by mitochondrial genes. Most of the methylation quantitative trait loci were located in the control region that is the most polymorphic region of the human mtDNA.

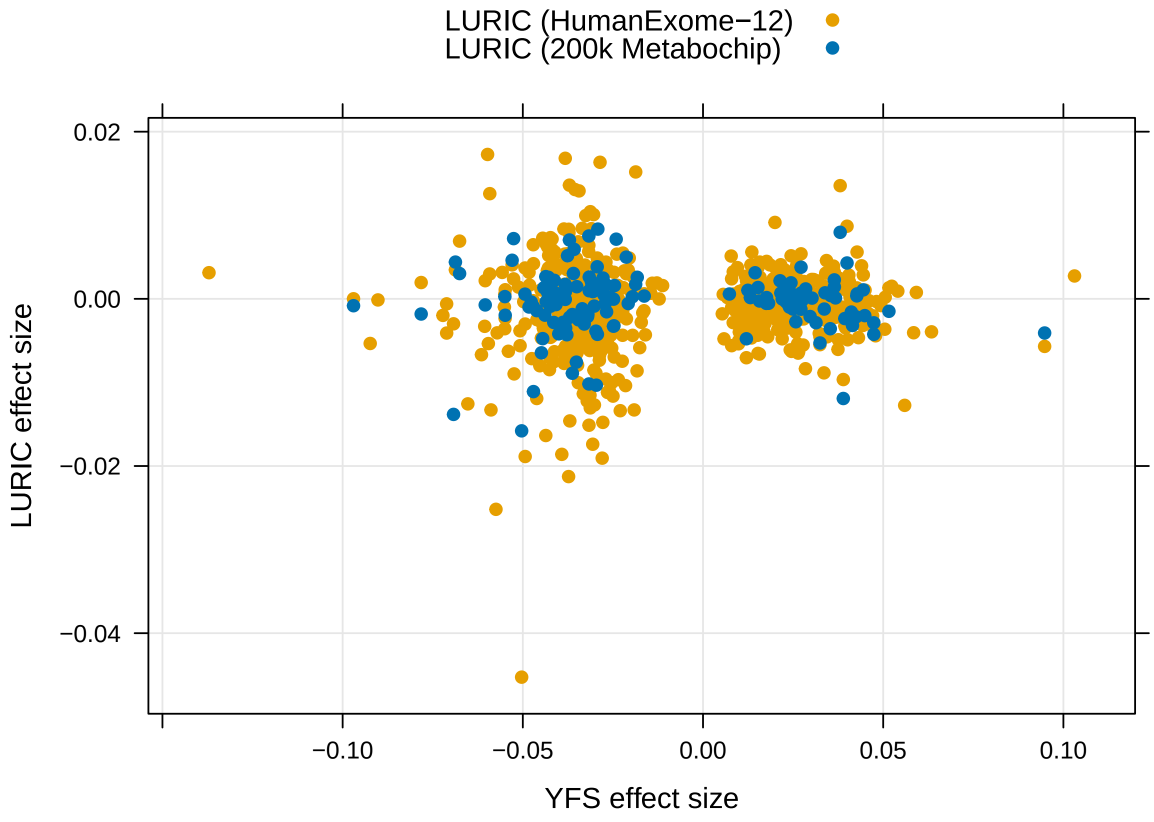

Figure S2: Discovery (YFS) and replication (LURIC) effect size estimates for 685 CpG–mtSNP pairs that were significant in the discovery phase and were available for replication.

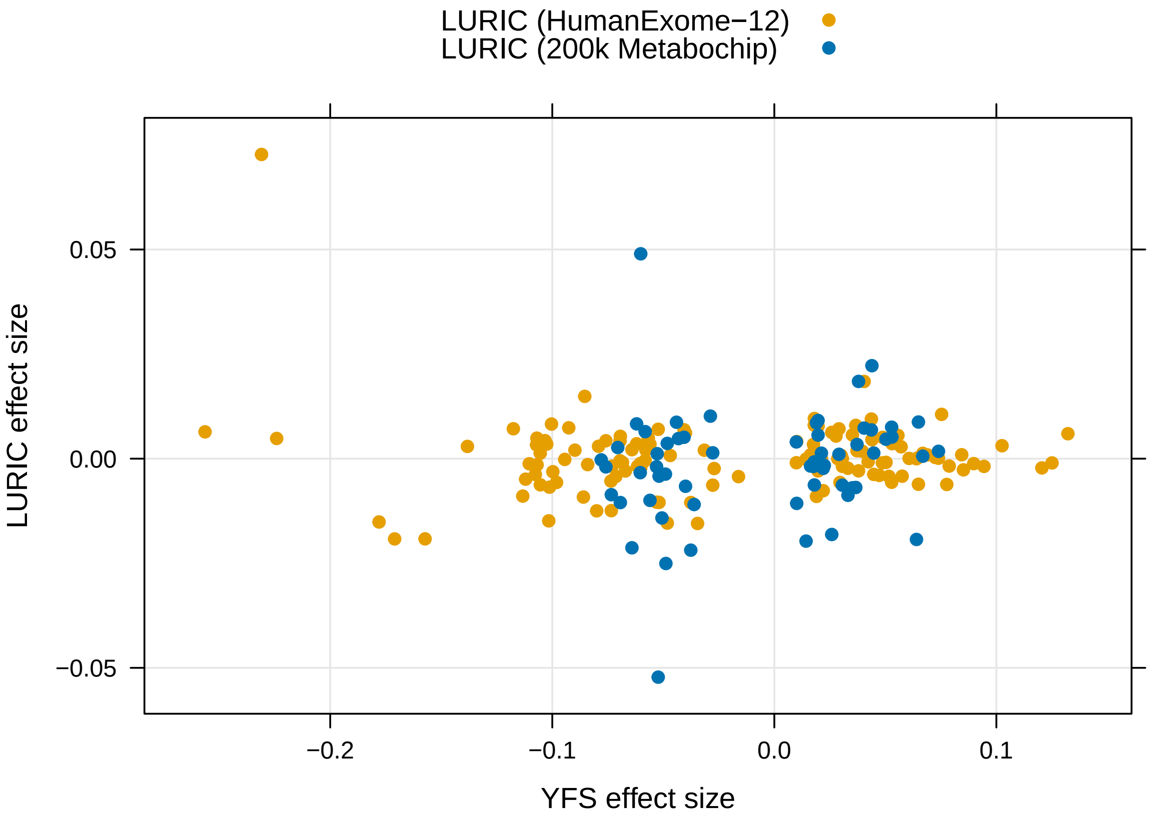

Figure S3: Discovery (YFS) and replication (LURIC) effect size estimates for 135 CpG–mtSNP pairs that were significant in the discovery phase in sex-specific analysis and were available for replication.

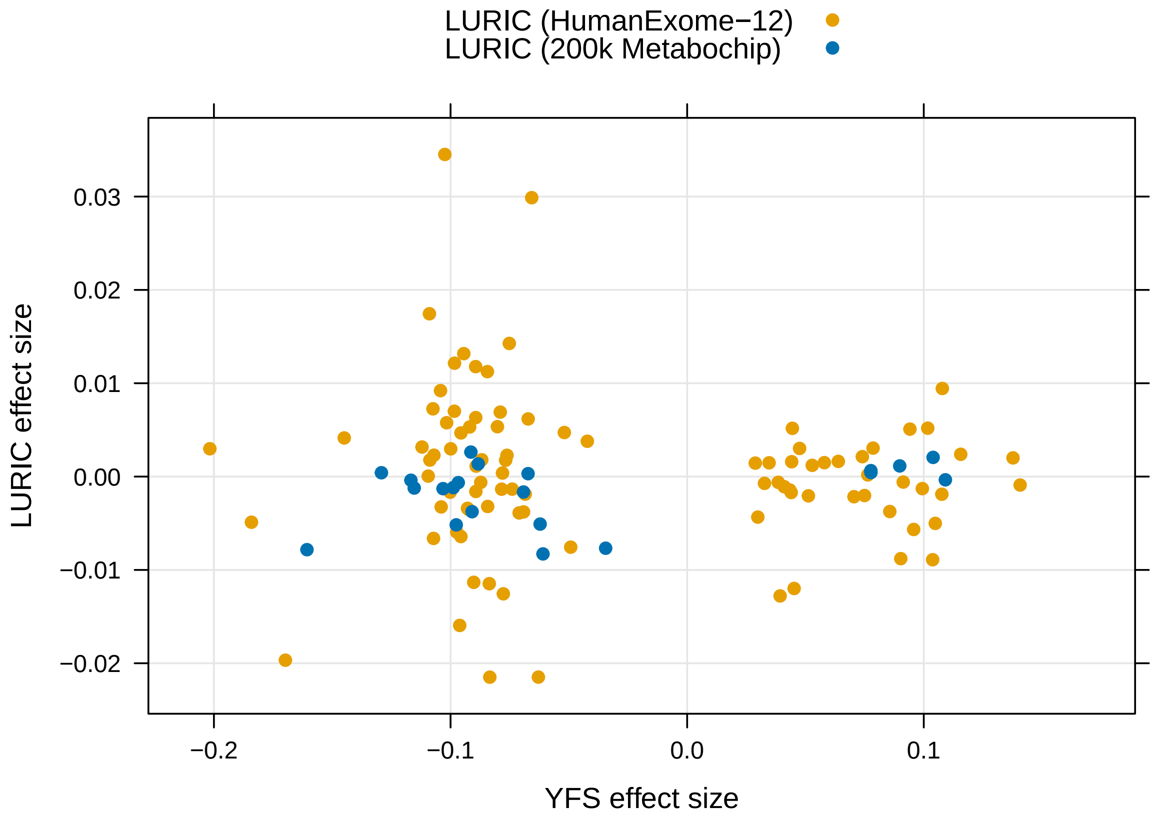

Figure S4: Discovery (YFS) and replication (LURIC) effect size estimates for 113 CpG–mtSNP pairs that were significant in the discovery phase in prediabetes-specific analysis and were available for replication.

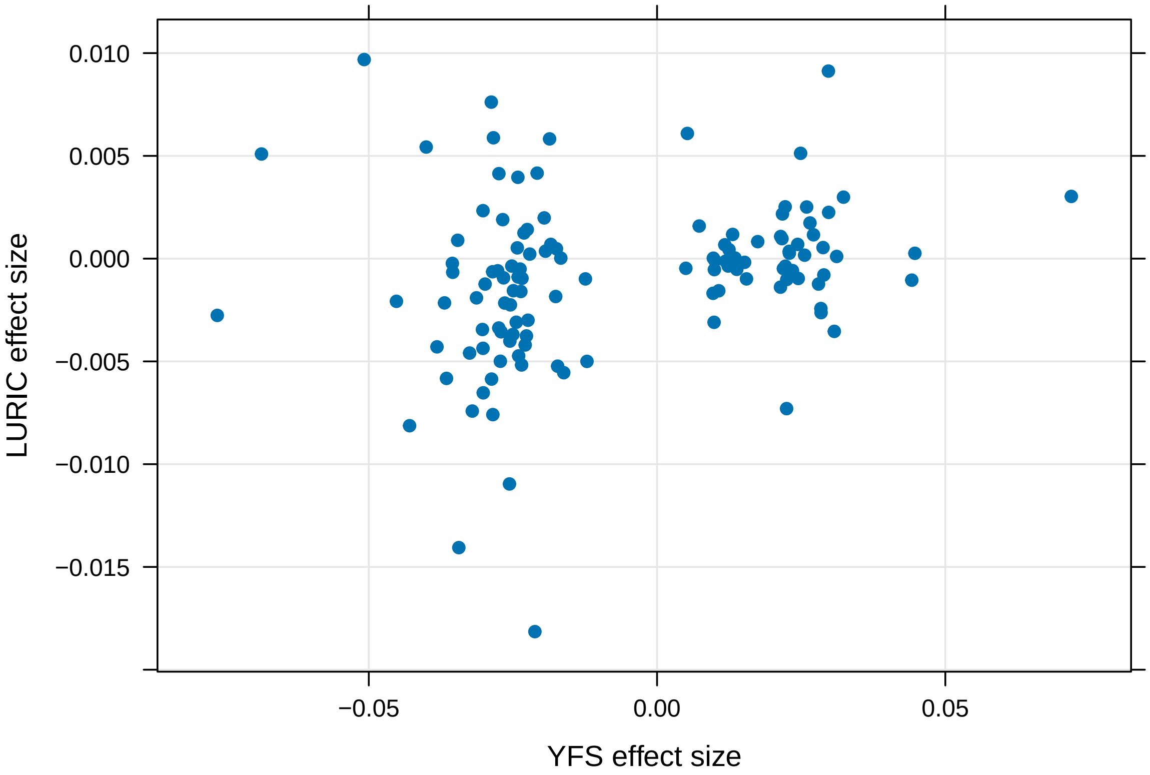

Figure S5: Discovery (YFS) and replication (LURIC) effect size estimates for 120 CpG–haplogroup pairs that were significant in the discovery phase and were available for replication.
