# Supplementary material for: Mitochondrial genome-wide analysis of nuclear DNA methylation quantitative trait loci
Source: Hum Mol Genet. 2021 Nov 19;31(10):1720–32. doi: 10.1093/hmg/ddab339 (PMC9122653; doi:10.1093/hmg/ddab339)
Supplement: Supplementary_tables_ddab339 [file supplementary_tables_ddab339.pdf]

Mitochondrial genome-wide analysis of nuclear DNA methylation quantitative trait loci

Supplementary tables

Supplementary Table 1. Haplogroup frequencies and the corresponding phenotype characteristics of the populations used in the haplogroup-specific analyses. Values are mean (SD) or n (%) for continuous and categorical variables, respectively.

Haplogroup	H	U	J	V	T	W	K	X	I
					YFS				
No. of participants	374 (43.3)	218 (25.3)	60 (7.0)	50 (5.8)	48 (5.6)	40 (4.6)	34 (3.9)	22 (2.5)	17 (2.0)
Age, years	42.0 (4.9)	42.3 (5.1)	40.8 (5.1)	41.6 (5.7)	42.1 (5.5)	42.0 (5.1)	41.4 (4.3)	42.2 (5.6)	42.6 (5.0)
Women	210 (56.2)	118 (54.1)	36 (60.0)	30 (60.0)	28 (58.3)	23 (57.5)	19 (55.9)	13 (59.1)	7 (41.2)
Body mass index, kg/m^2	26.7 (5.3)	26.5 (4.8)	26.7 (5.0)	26.9 (4.7)	25.2 (3.7)	26.9 (5.5)	26.8 (4.2)	28.2 (4.6)	27.2 (4.5)
Active smoker	50 (13.4)	32 (14.7)	9 (15.0)	4 (8.0)	2 (4.2)	7 (17.5)	7 (20.6)	3 (13.6)	3 (17.7)
Smokes once a week or more often but not daily	17 (4.6)	6 (2.8)	3 (5.0)	3 (6.0)	3 (6.3)	1 (2.5)	1 (2.9)	0 (0.0)	0 (0.0)
Smokes less often than once a week	14 (3.7)	7 (3.2)	1 (1.7)	3 (6.0)	2 (4.2)	1 (2.5)	1 (2.9)	4 (4.6)	1 (5.9)
Attempts to quit smoking	7 (1.9)	4 (1.8)	0 (0.0)	1 (2.0)	0 (0.0)	0 (0.0)	0 (0.0)	0 (0.0)	0 (0.0)
Has quit smoking	101 (27.0)	58 (26.6)	15 (25.0)	11 (22.0)	8 (16.7)	10 (25.0)	8 (23.5)	4 (18.2)	3 (17.7)
Has never smoked	185 (49.5)	111 (50.9)	32 (53.3)	28 (56.0)	33 (68.8)	21 (52.5)	17 (50.0)	14 (63.6)	10 (58.8)
					LURIC				
No. of participants	337 (33.8)	52 (5.2)	165 (16.5)	22 (2.2)	200 (20.0)	33 (3.3)	136 (13.6)	18 (1.8)	25 (3.5)
Age, years	63.1 (10.9)	62.1 (10.2)	61.5 (11.1)	63.6 (12.1)	62.0 (11.1)	61.4 (10.1)	63.6 (10.1)	61.2 (11.2)	62.2 (9.1)
Women	103 (30.6)	18 (34.6)	48 (29.1)	3 (13.6)	55 (27.5)	12 (36.4)	50 (36.8)	2 (11.1)	10 (28.6)
Body mass index, kg/m^2	27.8 (4.5)	27.3 (4.7)	27.2 (3.8)	27.3 (3.6)	27.7 (4.7)	28.0 (4.6)	27.7 (4.4)	26.6 (3.2)	27.7 (3.1)
Heavy smokers	53 (15.7)	5 (9.6)	19 (11.5)	4 (18.2)	31 (15.5)	8 (24.2)	19 (14.0)	3 (16.7)	5 (14.3)
Light smokers	21 (6.2)	5 (9.6)	10 (6.1)	1 (4.6)	15 (7.5)	1 (3.0)	16 (11.8)	2 (11.1)	4 (11.4)
Former smokers, quit < 10 years ago	46 (13.7)	3 (5.8)	24 (14.6)	2 (9.1)	29 (14.5)	3 (9.1)	15 (11.0)	4 (22.2)	10 (28.6)
Former smokers, quit ≥ 10 years ago	96 (28.5)	14 (26.9)	51 (30.9)	8 (36.4)	52 (26.0)	10 (30.3)	37 (27.2)	4 (22.2)	6 (17.1)
Has never smoked	121 (35.9)	25 (28.1)	61 (37.0)	7 (31.8)	73 (36.5)	11 (33.3)	49 (36.0)	5 (27.8)	10 (28.6)

Supplementary Table 2. List of tagging and the corresponding tagged mtSNPs in the YFS.

tagging mtSNP	tagged mtSNPs					
m.6045C>T	m.16051A>G	m.15907A>G	m.11732T>C	m.508A>G	m.6152T>C	m.13020T>C
	m.5390A>G	m.3720A>G	m.3116C>T	m.13734T>C	m.340C>T	m.11197C>T
	m.10876A>G					
m.6734G>A	m.250T>C	m.3990C>T	m.9053G>A	m.4529A>T	m.10034T>C	m.16391G>A
	m.8616G>T	m.203G>A	m.3447A>G	m.9947G>A		
m.15928G>A	m.11812A>G	m.14233A>G	m.4917A>G	m.8697G>A	m.10463T>C	m.15607A>G
	m.14905G>A	m.1888G>A	m.13368G>A			
m.3505A>G	m.12414T>C	m.5460G>A	m.8994G>A	m.5046G>A	m.15884G>C	m.1243T>C
	m.11674C>T	m.11947A>G	m.207G>A			
m.4024A>G	m.14365C>T	m.10044A>G	m.14582A>G	m.8269G>A	m.5004T>C	m.9123G>A
	m.3992C>T					
m.3480A>G	m.9055G>A	m.9698T>C	m.11299T>C	m.16224T>C	m.10550A>G	m.14167C>T
	m.1189T>C					
m.499G>A	m.4646T>C	m.5999T>C	m.11332C>T	m.15693T>C	m.6047A>G	m.14620C>T
m.153A>G	m.14470T>C	m.6221T>C	m.13966A>G	m.16255G>A	m.6371C>T	m.225G>A
m.12612A>G	m.489T>C	m.13708G>A	m.16069C>T	m.295C>T	m.462C>T	
m.12618G>A	m.7768A>G	m.150C>T	m.5656A>G	m.14182T>C		
m.4216T>C	m.16126T>C	m.15452C>A	m.11251A>G			
m.4580G>A	m.16298T>C	m.72T>C	m.15904C>T			
m.13928G>C	m.13827A>G	m.16114C>A	m.16526G>A			
m.10086A>G	m.12613G>A	m.9612G>A	m.4928T>C			
m.9477G>A	m.13617T>C	m.3197T>C	m.16270C>T			
m.11899T>C	m.3549C>T	m.7444G>A	m.16153G>A			
m.8448T>C	m.16293A>G	m.961T>G	m.13759G>A			
m.14564A>G	m.14482C>T	m.7013G>A				
m.12192G>A	m.9039G>A	m.8843T>C				
m.13780A>G	m.10238T>C	m.12501G>A				
m.186C>A	m.11191C>T	m.8715T>C				
m.10927T>C	m.16144T>C	m.7385A>G				
m.4452T>C	m.9066A>G	m.7309T>C				
m.11467A>G	m.12372G>A	m.12308A>G				
m.5263C>T	m.4639T>C	m.8869A>G				
m.9093A>G	m.9903T>C	m.11377G>A				
m.11719G>A	m.14766C>T					
m.5147G>A	m.930G>A					
m.2706A>G	m.7028C>T					
m.14793A>G	m.16256C>T					
m.14872C>T	m.2259C>T					
m.951G>A	m.16354C>T					
m.12630G>A	m.16271T>C					
m.16223C>T	m.12705C>T					
m.188A>G	m.185G>A					
m.4059C>T	m.1341C>T					
m.4769A>G	m.1438A>G					
